# Radial profile detection of multiple spherical particles in contact with interacting surfaces

**DOI:** 10.1371/journal.pone.0214815

**Published:** 2019-04-02

**Authors:** Johannes Waschke, Tilo Pompe, David Rettke, Stephan Schmidt, Mario Hlawitschka

**Affiliations:** 1 Max Planck Institute for Human Cognitive and Brain Sciences, Stephanstr. 1a, Leipzig, Germany; 2 Institute of Biochemistry, Leipzig University, Johannisallee 21-23, Leipzig, Germany; 3 Department of Chemistry, Heinrich Heine University Düsseldorf, Düsseldorf, Germany; 4 Faculty of Computer Science, Mathematics and Natural Sciences, Leipzig University of Applied Sciences, Leipzig, Germany; Texas A&M University College Station, UNITED STATES

## Abstract

Adhesive interactions of soft materials play an important role in nature and technology. Interaction energies can be quantified by determining contact areas of deformable microparticles with the help of reflection interference contrast microscopy (RICM). For high throughput screening of adhesive interactions, a method to automatically evaluate large amounts of interacting microparticles was developed. An image is taken which contains circular interference patterns with visual characteristics that depend on the probe’s shape due to its surface interaction. We propose to automatically detect radial profiles in images, and to measure the contact radius and size of the spherical probe, allowing the determination of particle-surface interaction energy in a simple and fast imaging and image analysis setup. To achieve this, we analyze the image gradient and we perform template matching that utilizes the physical foundations of reflection interference contrast microscopy.

## 1 Introduction

Adhesive interactions between deformable materials play an important role in technology as well as in biological processes, e.g. when cells interact with surfaces. In order to shed light on the underlying principles, adhesion phenomena need to be precisely quantified.

**Fig 1:**
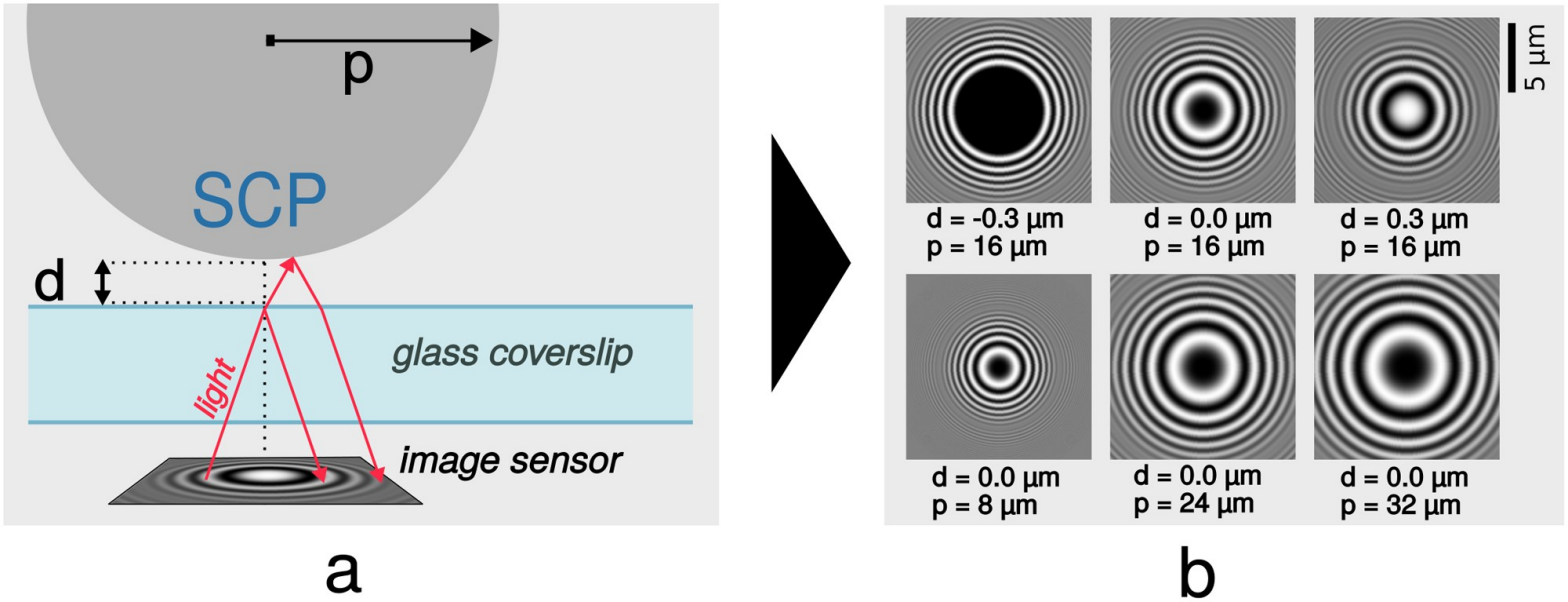
Image acquisition. Experimental setup (a), adapted from [6]. Light is emitted from behind the camera and light rays are reflected twice, leading to the characteristic interference patterns (b). Parameters *d* and *p* have a strong impact on the appearance of the ring-shaped patterns (simulation results).

Direct quantification of adhesion by means of a surface force apparatus or atomic force microscopy has provided valuable insights into the field of mechanobiology, bioadhesives and colloid science, to name just a few. While offering precise quantitative information on adhesive interactions down to the molecular level, these force-based techniques require considerable experimental effort. As a facile alternative, adhesion assays with soft polymer particles as probes (soft colloidal probes, SCPs) have been introduced to directly quantify adhesive interactions [1–3]. The method is based on determining the mechanical deformation of the SCP particles on a planar substrate by means of reflection interference contrast microscopy (RICM, see Fig 1). RICM as an imaging technique has long been successfully used to study the adhesion phenomena of cells, vesicles, and hard colloidal particles [4], since the underlying contacts can be visualized with nanometer-precision in the vertical direction using an optical microscope. When observing the adhesive contacts of SCP-particles with well-defined elastic modulus, the underlying adhesion energy *W*_*adh*_ can be quantified using the Johnson-Kendall-Roberts (JKR) model [3, 5]:

$$\begin{array}{c} W_{adh}=\frac{r_{c}^{3}\cdot \frac{4E}{3(1-v^{2})}}{6\pi p^{2}}\; \end{array}$$ (1)

where *p* is the SCP radius, *r*_*c*_ the radius of the contact area, *E* the Young’s modulus of the SCP and *v* the Poisson ratio.

In order to advance this method further, in the current work we establish software-aided analysis of the RICM-images—i.e. automated localization of adhesive contacts, detection of SCP radius and radius of contact area from the fringe pattern. Quantitative image analysis was already introduced in the early days of RICM. For example, Rädler and Sackmann analyzed the fringe patterns in RICM images and developed a theory that regards spatial distance between a sphere and substrate [6]. Later, an improved approach, which also considered the slope of non-planar surfaces, was proposed by Wiegand et al. [7]. With such a model as a foundation and with knowledge of the relevant parameters, it is possible to accurately simulate RICM profiles. Using non-planar image formation theory and numerical profile reconstruction, Contreras-Naranjo et al. [8, 9] developed more advanced algorithms showing improved profile reconstruction of unknown convex particle shapes. Clack and Groves [10] utilized the model of Wiegand et al. to create a library of 1250 synthetic templates for automated analysis of RICM images. The authors generated templates for particle sizes at height levels between 0 nm and 250 nm. Then they analyzed the microscope image via correlation filter to detect image regions having a high similarity to any of the templates. Related, but technically more demanding, methods, e.g. combining dual-wavelength RICM and atomic force microscopy, have made use of automated analyses to simultaneously measure force and absolute distance, while additionally relying on existing knowledge of particle size [11].

A quantitative analysis of SCP-surface interactions in biomedical and biosensor approaches with increased throughput relies on a large number of analyzed particles, simple and robust imaging, and reliable detection of variable object shapes and disturbing features. Various efficient image analysis algorithms have been developed for automated pattern detection in computer-aided image analysis. One such method is circle detection, which can be performed with the Circle Hough Transform [12]. More generally, many approaches exist to detect various kinds of objects in images. Common techniques include thresholding, edge-detection, watershed transformation, or region growing [13]. One quickly growing field is convolutional neural networks, which can be used for image classification and segmentation [14]. While the above-mentioned methods can detect the location of a profile, an analysis based on physical parameters (like radius, or spacing and number of peaks) is lacking. In addition, another major obstacle for automated profile detection is the variety of expressions of radial profiles, like in SCP-surface analysis. The shape of the profile depends on object size, object deformation, image scale, microscope setup, as well as the distance between probe and surface (Fig 1).

In this work, we aimed to develop a quick and efficient way to automatically process a high number of RICM images which should enable sampling of a large number of adhesive contacts, providing reliable quantification of adhesion energies by means of the JKR method. We considered the physical principles of the RICM image within the theory of Rädler and Sackmann [4] to reconstruct theoretical images and use them in an automated pattern matching algorithm. The experimental setup involves a glass coverslip with the adhered SCP placed on an inverted microscope setup, which can be functionalized in various ways for physiochemical, biomedical, or biosensing analysis [1, 15–18]. For high throughput, we use automated detection and profile analysis. Previous work demonstrated template matching as a means to detect the interaction of hard microparticles from RICM images [10]. Our method uses template matching as well, but we introduced a preliminary processing step that reduces the search space and thus vastly decreases the calculation time. Furthermore, we use a higher number of templates, which allows us to search for more heterogeneous expressions of fringe patterns (caused by varying SCP sizes and different contact radii due to deformation of the soft microparticles). Accordingly, our approach is able to detect SCPs and to intrinsically determine their size and contact radius in a highly efficient way involving GPU-accelerated calculation.

## 2 Materials and methods

### 2.1 Glass surface preparation

Sample interference patterns were obtained on hard glass surfaces. For the preparation of electrostatically repulsive and attractive model surfaces, glass coverslips (*ϕ*32 mm, Thermo Scientific, Germany) were placed in a Teflon rack and cleaned by sonication in double deionized water and ethanol (AppliChem, Germany) for 30 min each. Afterwards, chemical cleaning was performed to remove organic as well as particle contaminants from the surfaces. Therefore a mixture of 50 ml H_2_O_2_, 35% (Grüssing, Germany), 50 ml 25% NH_3_ aqueous solution (Merck, Germany) and 250 ml double deionized water was heated to 60°C on a hot plate and coverslips were left in the solution for 10 min. After rinsing twice with double deionized water, the coverslips were dried in a nitrogen stream and used as negatively charged (repulsive) surfaces.

Positively charged (attractive) surfaces were prepared by coating the clean glass slides with branched polyethylene imine (PEI) (average M_w_ ∼800 by LS, Merck, Germany). Here, a 20 mm solution of 3-aminopropyl-triethoxysilane (VWR, Germany) in a 1% (v/v) double deionized water / isopropanol mixture (AppliChem, Germany) was used for the introduction of amino groups to the glass surfaces. After a reaction time of 10 min, surfaces were washed thoroughly with isopropanol, dried in a nitrogen stream and annealed in a pre-heated oven for 60 min at 120°C. Subsequently, the glass slides were hydrolyzed for 2 h in double deionized water to remove excess and loosely bound silane. For further functionalization and stabilization of the silane layer, the amine coated slides were placed in a 240 mm succinic anhydride solution in THF (Grüssing, Germany) and allowed to react for 1 h. Following 2 washing steps, 1.5 ml of a 20 mm 1-Ethyl-3-(3-dimethylaminopropyl)carbodiimide (EDC) / 50 mm N-Hydroxysuccinimide (NHS) solution (Carbolution Chemicals, Germany; Merck, Germany) in HEPES buffer (Carl Roth, Germany) 100 mm, pH = 7.0 was pipetted on top of each glass slide. After 15 min of activation, 1.5 ml of a 4 mm PEI solution in the same buffer was added to the slides and the reaction was allowed to proceed for 1 h. Finally, the coated surfaces were rinsed three times in HEPES buffer.

### 2.2 Particle preparation

SCP particles were synthesized as described previously [17]. Briefly, 50 mg poly(ethylene glycol) diacrylate (Mn 6000 Da, Sigma Aldrich, Germany) and 1 mg of the photoinitiator Irgacure 2959 (Sigma Aldrich, Germany) were added to 10 ml 1M sodium sulfate and vortexed until microscopic droplets were formed. The dispersion was photopolymerized using a Heraeus HiLite UV curing unit (Heraeus Kulzer, Germany) for 90 s. Next, the PEG SCPs were grafted with crotonic acid (Sigma Aldrich, Germany) as described earlier [15]. In short, water was exchanged by 10 ml ethanol, then 250 mg benzophenone and 1.5 g crotonic acid were added. Subsequently, the mixture was flushed with nitrogen for 30 s before irradiating with UV light for 900 s. The resulting SCP particles were then washed with ethanol and PBS three times each. This synthesis resulted in SCPs with a Young’s elastic modulus of approximately 40 kPa as characterized by scanning probe spectroscopy, for details see [17].

### 2.3 Reflection interference contrast microscopy and bright-field imaging

Cleaned or PEI-coated cover glasses were placed in a sealed PTFE-ring and each surface was covered with 1 ml of a 10% ethanol (AppliChem, Germany) HEPES-buffer mixture pH = 7.0. 100 μl of a suspension containing COOH-functionalized particles (section 2.2) was added dropwise afterwards. After a period of 15 min, sedimentation of the particles was completed and the probes and their corresponding radial profiles were imaged using an inverted microscope (Olympus IX73, Germany) with an integrated halogen lamp for bright-field microscopy. To obtain the respective interference reflection patterns, samples were illuminated by a monochromatic 530 nm collimated LED (M530L2-C1, Thorlabs, Germany). An Olympus 60x, NA (numerical aperture) 1.35 oil-immersion objective (UPlanSApo 60x 1.35 oil, Olympus, Germany) was used in concert with a quarter waveplate (WPMQ05M-532, Thorlabs GmbH, Germany), placed on the microscope’s breadboard between objective and sample, as well as additional polarizers to avoid internal reflections [19]. Images were captured with a monochrome CCD camera (DMK 23U274, ImagingSource, Germany) using *μ*Manager microscopy software. All datasets were recorded applying an exposure time of 50 ms and stored in tagged image file format (tiff). Besides the methods explained in the following sections, no image processing was performed.

### 2.4 RICM model

Reflection interference contrast microscopy (RICM) is suitable to measure nanoscale distances between a planar transparent surface and another object, like the aforementioned SCPs. The main idea is based on the fact that a light ray is reflected (at least) twice—once from the planar transparent glass surface, once from the particle—and the phase difference between the two interfering rays translates into increased or decreased intensity on the image sensor (Fig 1). The phase difference is basically a geometrical problem and a basic model concerning this experimental situation was established by [6], see also details to other model extensions and approaches in the introduction. The intensity along a profile’s radius *r* of a spherical hydrogel particle in an aqueous solution (with parameters from Table 1 and *h*(*r*) from Eq 5) can be calculated by

$$\begin{array}{c} p_{template}\left(r\right)=\frac{\sin y}{y}\cdot \cos(2kh\left(r\right)\cdot (1-\sin^{2}\frac{\alpha }{2})+\theta ) \end{array}$$ (2)

$$\begin{array}{c} \;\text{with}\;\alpha =\sin^{-1}\frac{INA}{n}\;\text{and}\;k=\frac{2\pi n}{\lambda }\;\text{and}\;y=2kh\left(r\right)\cdot \sin^{2}\frac{\alpha }{2}\;. \end{array}$$ (3)

**Table 1:** Overview of parameters.

	**Template Profile Parameters**	**Value**
*u*	Pixel length [μm]	0.067
λ	Wavelength [μm]	0.53
*INA*	Numerical aperture of illumination	0.67
*r*_max_	Radius of generated templates [pixel]	100
*n*	Refraction index	1.332
*θ*	Phase shift	*π*
*dec*	Decay	[0, 1, …, 10]
*d*	Particle height [μm]	[-0.45, -0.44, …, 0]
*p*	Particle radius [μm]	[10, 11, …, 30]
	**Sampling and Detection Parameters**	**Value**
*m*	Number of radial samples per position	90
*c*	Percentage of pixels to remain after pre-processing (in%)	1
*a*	Minimum amplitude of the local profile *p*_mean_	0.05
*t*_1_	Minimum correlation of *p*_mean_ to best template	0.9
*t*_2_	Minimum average correlation between *p*_mean_ and the radial samples *p*_*i*_∀*i*	0.5

The upper part shows parameters for the template generation. Single values are constant for all templates; the values provided as ranges define the search space. The lower part contains parameters for the search process and the last three rows define constraints that every detected profile must fulfill.

The applied basic RICM theory is known to exhibit certain deviations in exactly describing the intensity distribution, but, as we will show later in the validation section, is appropriate to provide a simple and fast approach to analyze SCP-surface interaction. Intensity deviations for outer peaks can be handled using an empirical exponential decay, as suggested in previous work [4]:

$$\begin{array}{c} p_{\text{template}}{\left(r\right)}^{\prime }=e^{\frac{-dec\cdot r^{2}}{r_{max}}}\cdot p_{\text{template}}\left(r\right)\;. \end{array}$$ (4)

### 2.5 Implementation details

We implemented the algorithms as a standalone software tool with the Qt framework (C++). The calculation of the template correlation is pixel-wise independent and it involves many numerical computations, which is the reason why we utilized OpenCL for a GPU-driven parallel calculation. The evaluation was performed on a desktop computer (Intel Core i7 4770, AMD R9 280X Graphics Card, 16GB RAM).

## 3 Algorithm development and results

For automated RICM image analysis, the variety of profiles hampers the utilization of most detection algorithms, but since the profile shape follows physical laws, we can harness the physically predicted profiles to detect and analyze the shape of adhered objects. These template profiles are used to perform template matching over promising image regions. As an additional benefit, we can also derive the probe size and the contact radius from the best-matching template. However, template matching is a computationally expensive procedure and thus analyzing the full image would lead to relatively long calculation times. Prior to the template matching, we exclude image parts from the search space by analyzing their gradient orientation.

Based on these general considerations we developed an algorithm which follows three main steps:

- Pre-selection based gradient orientation
- Template matching
- Data extraction and calculation of results

**Fig 2:**
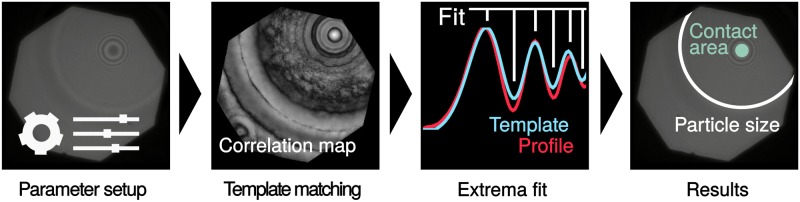
General workflow. Processing steps of an (experimental) image containing one particle. The result includes the particle position, particle size, and its contact area.

**Fig 3:**
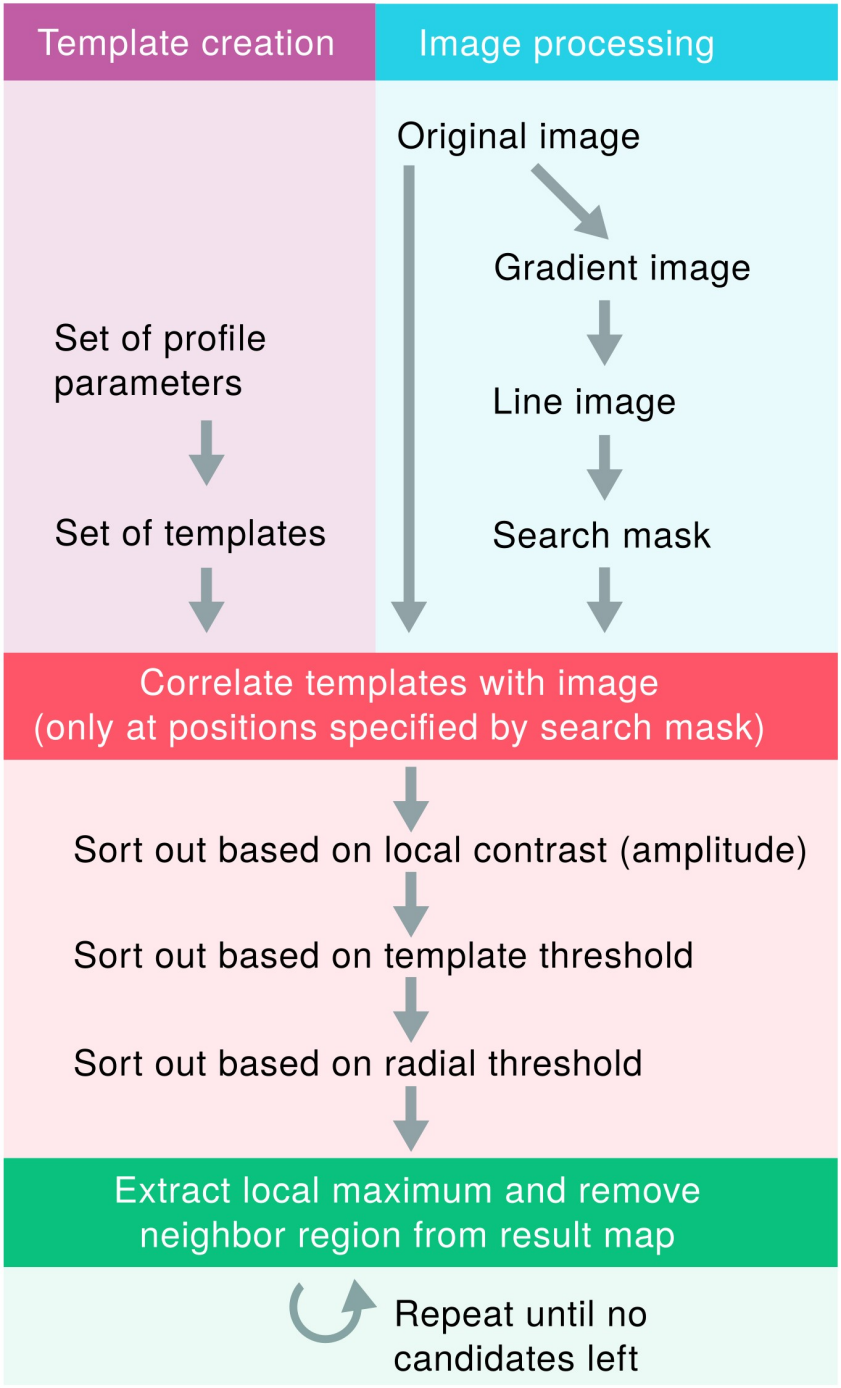
Workflow of the detection process. The processing steps of template creation and image processing.

The general workflow of the image analysis algorithm and the detailed workflow of the detection is additionally illustrated in Figs 2 and 3.

### 3.1 Object distance and particle size

**Fig 4:**
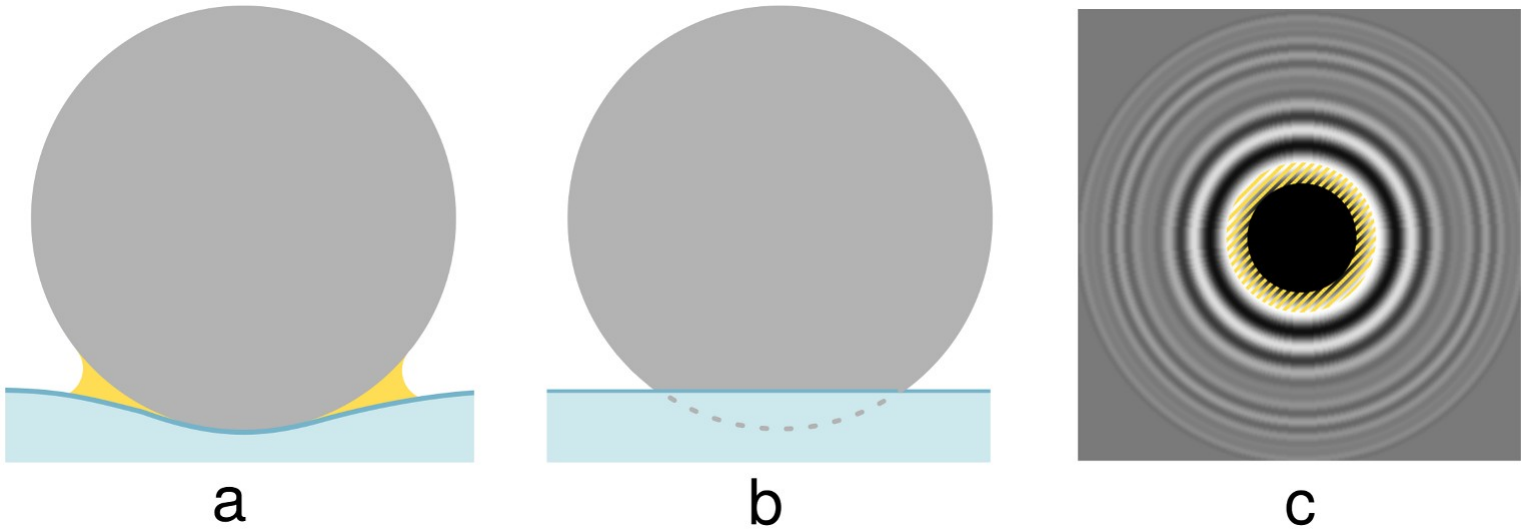
Geometric model assumptions. Deformations or menisci (yellow) can occur and vary depending on material properties (a). We assume a simpler geometric model (b), which is independent of such material properties but, however, could lead to deviations along the border of the contact area (hatched yellow area in the simulated image (c)).

Eq 2 requires knowledge about the distance between the surface and the SCP object at any radius position *r*. Though the particles are in general spheric, recent publications describe neck-like deformations along the border of the contact zone [20] or suggest a water meniscus [9] around the particle (Fig 4). These effects depend e.g. on the elasto-capillarity of the surfaces and result in image artifacts along the contact border. However, since the concrete shape of the artifacts is hard to predict, and because of their relatively little impact on only minor regions in the image, we assume the SCPs to be perfectly round and ignore any deformations of the probe. These assumptions are valid, as seen from the results below and earlier reports [1]. For touching surfaces (*d* ≤ 0), we set *d* ≔ 0 which crops the sphere. Thus, with parameters from Table 1 we calculate the height with

$$\begin{array}{c} h\left(r\right)=\max(0,d+p-\sqrt{p^{2}-r^{2}})\;. \end{array}$$ (5)

### 3.2 Pre-selection based on gradient orientation

**Fig 5:**
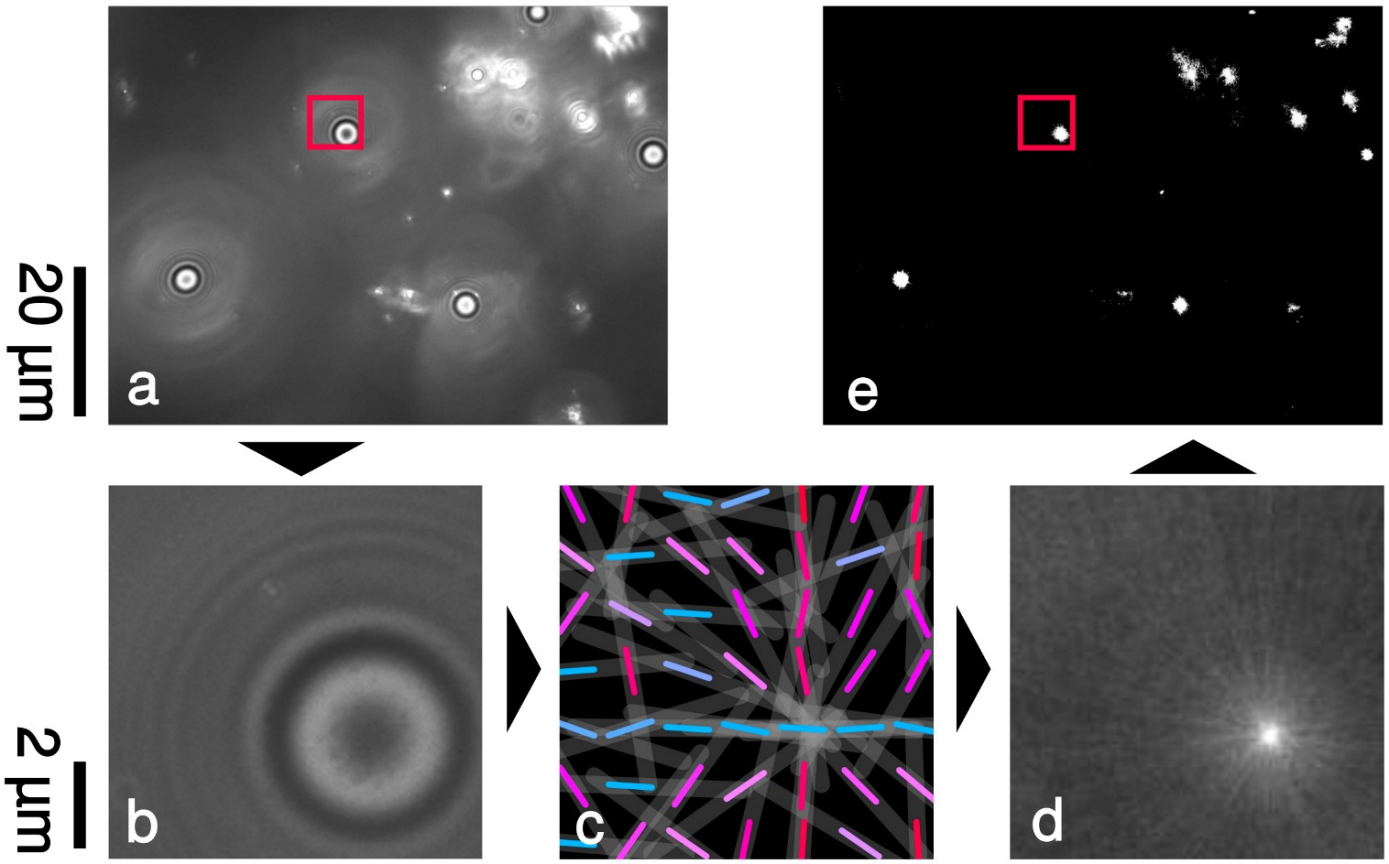
Increasing performance by reducing the search-space. A pre-processing step aims to reduce the search space. To achieve this, the gradient is calculated (glyphs in Fig c, with color-coding based on their orientation) and lines are drawn along the gradient for every pixel (line image). Overlapping lines lead to intensity peaks in the center of radial profiles (d). These peaks are extracted with thresholding and they form the search space for the template matching process (e). Images a and b are experimental results (acquired as described in section 2.3) and their contrast was enhanced for presentation purposes.

To reduce the calculation time of the template matching (see next subsection), we attempt to exclude less interesting image parts from the search space. We utilize the Sobel operator to calculate the image gradient. For every image position with a non-zero gradient, we draw a straight line along the gradient orientation into a new image (*line image*, see Fig 5c and 5d). The length of the line is chosen according to the expected profile radius of *r*_max_ pixels. Caused by the circular shape of the profiles, lines will cluster in the center of profile positions. These clusters are visible as bright spots in the line image. We then select the brightest *c* percent of pixels and restrict the template matching to these positions (see Fig 5). Since the intensities at the center of spherical objects in the line image are more than exponentially higher than surrounding pixels, a high number of pixels can be omitted without losing coverage of important image regions around the profile centers. Consequently, the reduction of the search space to e.g. 1% of the image increases calculation speed up to a factor of 100.

### 3.3 Template matching

We create templates on basis of the model from Eq 2 and the distance function from Eq 5. The parameters are mostly constrained within an experiment and we can use these limits to define a minimum search space. We pre-calculate the profiles for a number (e.g. ∼ 8 000) of possible combinations.

For the actual detection process, we consider all candidate pixels provided by the pre-processing step. At each such pixel, we sample starting from a center position (*x*, *y*) along radius *r* = 1 … *r*_max_. We calculate the average (*p*_mean_) of all profiles *p*_*i*_. With this process, noise is reduced and image positions with radial symmetry are favored since their amplitude is maximum. With *I*(*x*, *y*) being the intensity at image coordinates (*x*, *y*), the sampling steps are defined by

$$\begin{array}{c} p_{i}\left(r\right)=I(x+r\cdot \sin(\frac{2\pi i}{m}),y+r\cdot \cos(\frac{2\pi i}{m})) \end{array}$$ (6)

and the average profile is

$$\begin{array}{c} p_{\text{mean}}\left(r\right)=\frac{1}{m}\sum_{i=1}^{m}p_{i}\left(r\right)\;. \end{array}$$ (7)

As the next step, we calculate the Pearson correlation coefficients between *p*_mean_ and all pre-calculated templates. We save the index of the template with the highest similarity and we also note the respective correlation in a map.

We also make use of additional constraints to reduce false positives. One problem is the fact that correlation as comparison metric ignores the amplitude. Our solution is to define a minimum amplitude *a* that a certain profile *p*_mean_ from Eq 7 should contain after it was sampled from the image (e.g. 5% of the whole intensity range). Profiles with lower contrast are omitted.

To further reduce false positives, we introduce a threshold for circular correlation (*t*_2_). We only consider positions for which all samples *p*_*i*_ have at least a certain mean correlation *t*_2_ to *p*_mean_ and thus we exclude asymmetrical image parts. This step is applicable thanks to the fact that our objects of interest always have a spherical shape.

### 3.4 Extraction of matches and contact radius

**Fig 6:**
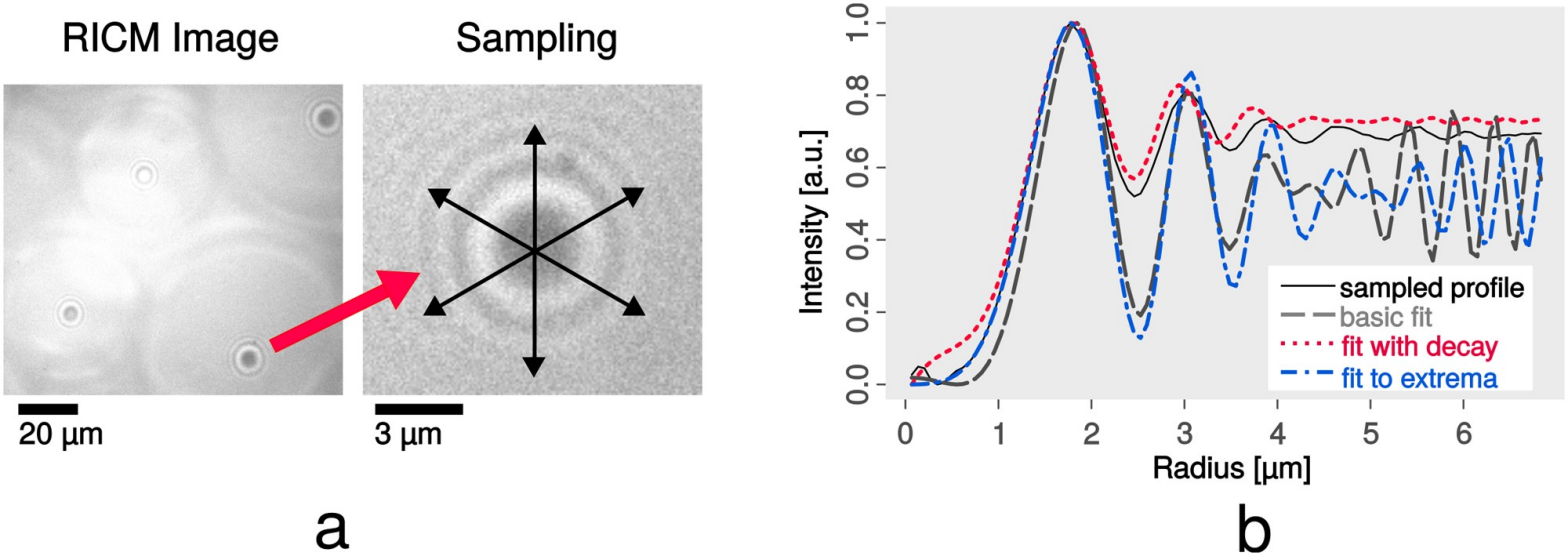
Sampling and fitting of profiles. Fig a shows a RICM image and sampling at an exemplary position (brightness increased for presentation). Fig b demonstrates the fitting procedure of the obtained profile. Our basic template fit for this example shows good Pearson correlation of 0.91, however, adding an exponential decay for higher radii improves correlation to 0.99. To determine particle size, we fit the template regarding optimized extrema overlap (note the better match especially of low peaks for radius positions 5 μm and higher).

The result of the previous calculation is a map of correlations and information of the respective best-matching template. We search for the maximum value in the map. Next, we save the respective coordinates and we store the parameters of the best-matching template. Finally, we apply a consecutive template matching step at this position. This time we ignore the amplitude but we focus on a good matching of the extrema (Fig 6), since they directly relate to particle size. We define similarity by counting the number of positions with the same slope orientation as the sampled profile has (both simultaneously falling or rising) and normalize by *r*_max_. This metric holds the percentage of the template and the measured profile running in synchronism. After extraction, we set a neighborhood of radius *r*_max_ around the current match to zero and we repeat the search process on the updated map. We stop the extraction when all positions of the correlation map contain a value less than *t*_1_ (and thus no image position shows sufficient similarity to any of the templates), or when a maximum number of iterations is reached. The radius *r*_*c*_ of the contact area can be derived from the particle radius *p* and particle height *d*, which are associated with the respective best-matching template, by calculating the intersection of the sphere and the coverslip plane:

$$\begin{array}{c} r_{c}=2\sqrt{2pd-d^{2}}\;. \end{array}$$ (8)

Note, due to the periodicity of the cosine (right term of Eq 2), it is hardly possible to determine the absolute height of the particle. We can only compute the particle height relative to the phase of the light

$$\begin{array}{c} d_{relative}=offset+i\frac{\lambda }{2n(1-\sin^{2}\frac{\alpha }{2})}\;, \end{array}$$ (9)

where *offset* equals the parameter *d* of the best-matching template and *i* is an integer. For all *i* ≥ 0, the frequency of the rings is identical. The amplitude (left term of Eq 2), however, is dependent on the absolute height, but its effect on the image is relatively low. This makes it difficult to determine *i* (with our simple and cost-efficient experimental setup) and thus leads to an ambiguous solution for the absolute particle height. We could avoid this problem by an advanced experimental setup involving dual wavelengths [21]. However, we are only interested in touching surfaces of interacting functionalized SCPs and surfaces in physiochemical, biomedical and biosensing applications, hence we can assume *i* ≤ 0. For negative heights, the profiles additionally show a distinct uniform center (the contact area) and, thus, there is no ambiguity in the appearance of the profiles.

### 3.5 Evaluation

We implemented the method in a software tool that allows the user to load a series of images, define suitable parameters (Table 1) and start batch processing of all images. We support the user by providing parameter estimations for manually defined sample positions and by providing presets. The calculation speed grows linearly with the number of templates, the image size, the radius of a profile, and the number of samples *m*. In addition, a constant time span is required to load data and to store results. The calculation speed for a full image—without pre-processing—is roughly 20s for one image (1 600 × 1 200 pixels) with a number of 8 000 templates and a maximum radius of 100 pixels. However, enabling pre-selection of image regions (as explained in section 3.2) leads to an overall calculation time of less than 2.5s per image (570s for a test stack of 265 images).

**Fig 7:**
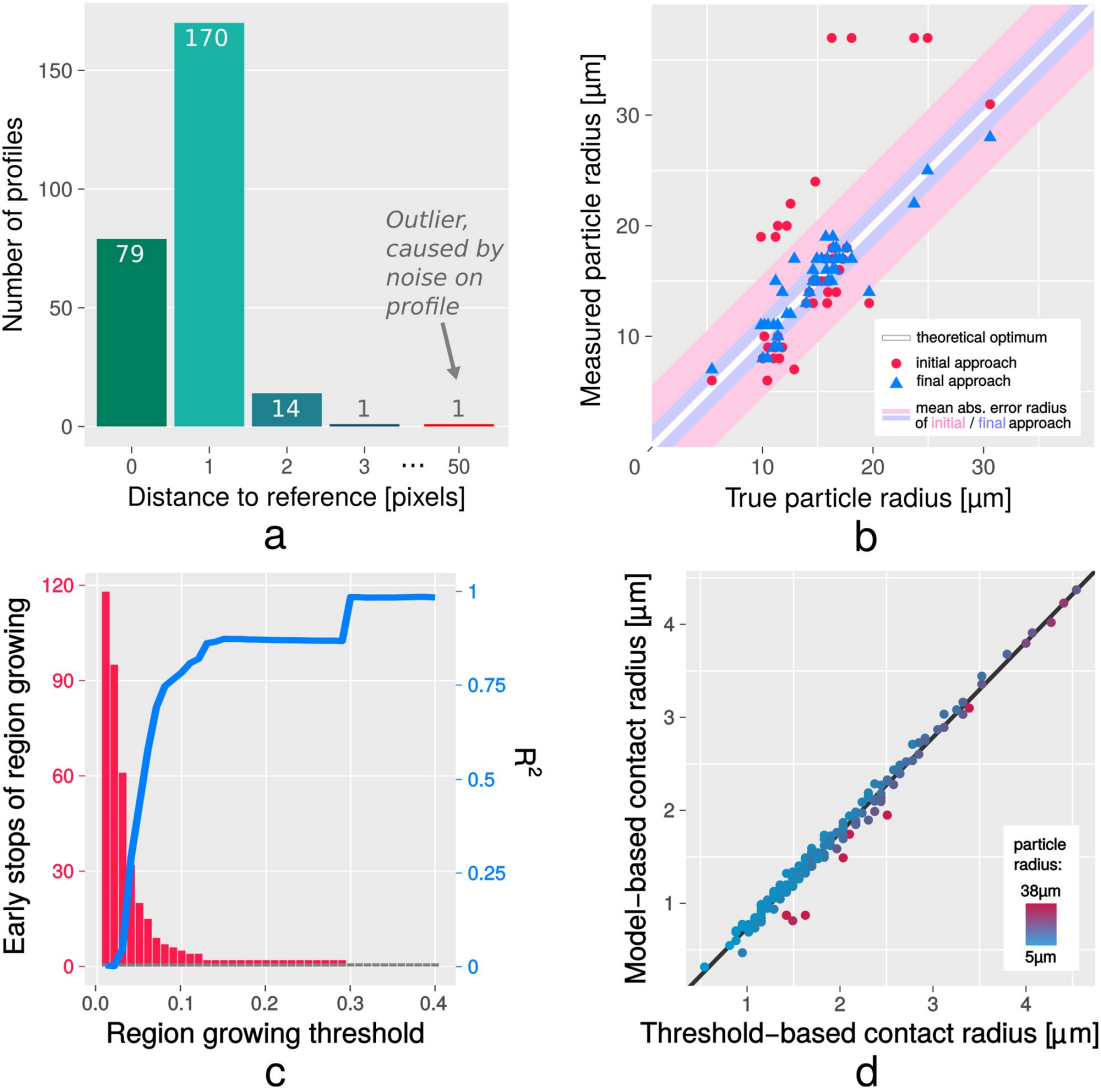
Evaluation of particle detection, particle radius and contact radius. The histogram (Fig a) displays the deviation for the location of the profiles compared to manually labeled reference data (total of 265 samples). The scatterplot (Fig b) shows results for particle radius determination compared to manually labeled reference data (total of 47 samples). It can be seen that the optimized version, in which particle size is determined on basis of a second extrema-focused matching, works notably better (mean absolute error 1.3 μm) than our naive approach (mean absolute error 3.8 μm). The bottom row shows the results for the comparison between the model-based contact radius and the reference data created by region growing. Plot c presents an evaluation for various region growing thresholds between 1% and 40% of the profile amplitude. Plot d shows detailed results for the lowest threshold (30%) that was free of erroneous stops during region growing, and the regression line (*R*^2^ = 0.9856). The plot reveals an overestimation of the contact radius by the threshold-based approach, which is a consequence of the relatively high threshold. Lower thresholds would lead to earlier stops (and thus smaller radii) but also to erroneous stops (see Fig c). Further details are given in section 4.3.

Starting the calculation with adequate search space usually results in a Pearson correlation of more than 0.95 at profile positions, which is in most cases notably higher than any other profile-free image part. For relatively complex profiles (e.g. involving intensity drops at inner and outer radius positions) the highest correlation might also decrease to 0.8. We used test data for which we initiated the detection process session-wise. For example, images with a constant microscope setup were analyzed in one batch. For proper sampling of the profiles, it is vital to know their accurate center position. To verify the localization of profiles, we used a test data stack of 265 images of SCPs and PEI-coated (attractive) surfaces. All profiles have been detected and the average distance to the manually defined reference position was 1.08 pixels (see Fig 7a). However, false positives are occasionally caused by round objects or air bubbles that are also present in the data. In our test case, 11 false positives have been detected, but they were never marked as the first choice since their template correlation was comparably low. Increasing the radial correlation threshold would reduce the number of false positives but also leads to false negatives. In summary, we prefer to use a tolerant threshold that reliably detects all present profiles. False positives can be quickly deleted in a manual review step.

To verify the particle size, which we derived from the parameters of the best-matching template, we acquired another test data of SCPs on PEI-coated (attractive) surfaces showing 47 particles. The test data includes bright-field data (as reference) in which we could manually measure the size of the SCPs. The validation of the particle diameter shows a mean absolute difference to the reference data of 2.6 μm for particle diameters from 11 μm to 62 μm (see Fig 7b for details).

Evaluation of the contact radius faces the problem of a missing direct measurement as reference. However, the contact radius can also be estimated from the size of the flat dark area in the center of a profile: we determined the contact radius in the average profile *p*_*mean*_ based on region growing. We started with the center position of the profile and iteratively selected neighbor positions that showed similar intensity to the center position. The stopping criterion for this process was an increase of the intensity by a certain percentage of the profile’s overall amplitude. The concrete choice of the threshold is a trade-off between sensitivity towards noise (too strict threshold) and overestimation of the contact radius (too tolerant threshold), therefore we analyzed the result of 40 uniformely distributed thresholds in the range from 1% to 40%. We then manually reviewed the results and chose the lowest threshold for which region growing never failed, which was a value of 30%.

Our test data consists of 205 images containing in total 184 adhering particles (hence, for these particles the best-matching template implied *d* ≤ 0). We describe the relationship between the result *c*_*model*_ of the model-based approach and the result *c*_*intensity*_ of the intensity-based reference by the linear relationship

$$\begin{array}{c} c_{model}=a\cdot c_{intensity}+b\;. \end{array}$$ (10)

For the threshold of 30%, the function *lm* (linear model) in R Statistics determined *a* = 1.0247 and *b* = −0.2890 μm (adjusted *R*^2^ = 0.9856), which shows a strong linear relationship of our approach to the intensity-based reference data (see section 4.3 and Fig 7c and 7d for details).

## 4 Discussion

In this section we want to first discuss methodical aspects and explain the merits of the chosen techniques compared to possible alternatives. Subsequently, we provide justification for the simplifications and discuss the evaluation presented in section 3.5.

### 4.1 Methodological considerations

We have developed a method that generates templates based on an established physical model. We use a pre-processing step to accelerate the calculation and we apply Pearson correlation to compare the templates with the microscope image. A consecutive fit based on the profile slope improves the estimation of the particle radius considerably.

The method we present relies on knowledge of a very specific use-case, but with a broad applicability in science and engineering. For example, the method may be applicable to physiochemical interactions of soft hydrogel particles and mimics of living cells with interacting surfaces [22], determination of protein adsorption energies on materials surfaces [23] or biosensing approaches [15, 16, 18]. Naturally, a solution based on a more general and re-usable approach would be preferred, however many such approaches we examined lead to noisy results or miss profile occurrences. For example, one technique we designed focused only on statistical properties of the set of profiles *p*_*i*_ (*i* = 0…*m*) from Eq 6. We searched for positions of simultaneously low variance between all the sampled profiles *p*_*i*_ and high variance inside the averaged profile *p*_mean_. This approach matched positions with high rotational symmetry and strong contrast. However, this simplified method produced less predictable special cases, and the problem of profile evaluation remained.

Our images contained brightness shifts (vignetting) and could be noisy or cluttered due to their application on biofunctionalized surfaces and high-throughput tasks, which impaired results obtained by thresholding or edge detection. The variety of frequencies of the rings and thus the strength of the edges made it particularly difficult to detect all rings of an image with a single edge detection setup. Usually, edge detection would only be able to detect thin or thick edges separately. Furthermore, we utilized Circle Hough Transform [12] to detect rings formed by intensity peaks of the profile, but the result was sensitively depending on the chosen parameters. Even in the best case only a few rings with high contrast could be found, which might be related to the above-mentioned difficulties in edge detection. This would lead to inaccurate results since the frequency and thus detection of every single ring is of high importance. While classical template matching with manually defined templates could be an option, the circular patterns differ strongly in appearance, and the variance in the frequency of intensity peaks makes it cumbersome to manually define a profile library. Another popular option has been machine learning, which we initially considered but we lacked sufficient training data. The number of parameters which affect the shape of the profile is comparably high and hence many combinations of training cases would be required. Furthermore, the evaluation of the detected profile would likely require for a second processing step and thus we rejected that approach.

Thus, while other promising techniques may exist, we reasoned that a positive outcome was more likely by trying to advance existing techniques that can already represent the issue with a physical model. With developing theories on reflection interference contrast microscopy, future updates of the model could be implemented with little effort.

### 4.2 Simplifications

The model we use as a basis for template calculation involves simplifications. We consider the SCP to be perfectly spheric (see Eq 5), even for the case of touching surfaces (*d* ≤ 0), although deformations of the SCP have to be present [20]. This leads to minor artifacts on the boundary between contact area and non-contact area, since the transition is very sharp. These artifacts could be addressed by a more detailed model in the future, although the effects on the outcome are considered to be of minor impact, as our validation results show. Furthermore, Eq 2 ignores changes in the angle of the light rays that were reflected from the SCP surface. An analytical solution seems to be unknown [19].

### 4.3 Evaluation

An important metric is the correctness of the position of the detected templates. Wrong coordinates would lead to an asynchronous radial sampling. This causes increased variance between the samples *p*_*i*_ and eventually a distorted profile *p*_*mean*_. Consequently, the parameters derived from this profile would be inaccurate as well. Clack and Groves [10] verified their precision by checking if their matches were precisely placed in the center of symmetry of the measured profile, and if they were stable for repeated measurements of immobile particles. We decided to use manually labeled reference data, since in several cases artifacts (e.g. bubbles) lead to asymmetric patterns. Our result shows a mean deviation of 1.08 pixels (∼ 0.07 μm), which seems to be very accurate.

The particle size is probably the most challenging parameter here (it suffers most from inaccurate templates), therefore the deviation of 1.3 μm to the reference data is relatively high. However, other methods only work with already known particle sizes from other analytical techniques [10], but with our method a separate measurement of the particle size can be omitted. The results for this parameter could probably be improved with models that allow a better fit to the underlying RICM image.

The contact radius is often visible in the RICM images as a flat dark area in the center of the profile, which makes it relatively easy to define by basic image processing. A challenge for region growing are the noisy center positions of *p*_*mean*_. The radial averaging has only little effect for small radii, and most extreme, at radius position 0 all profiles *p*_*i*_ sample from the same pixel of the image. As expected, the number of failed region growing attempts was high for low thresholds. Therefore we chose a threshold of 30%, which resulted in valid region growing for all test samples. Nevertheless, all thresholds of at least 10% indicated a very strong similarity of our approach compared to the results of region growing. Due to the tolerant threshold, region growing usually stopped relatively late, which results in an overestimation of the contact radius (as can be seen in the offset *b* = −0.2890 μm from the linear regression). An evaluation based on a lower threshold led to *b* closer to 0 μm, but more outliers led to a worse *R*^2^. Our reference data contained particles from 5 μm to 38 μm (mean: 13.84 μm). The absolute error between our results and the reference data (mean: 0.0643 μm) depended moderately on the particle size (Pearson correlation of 0.5492, which means that deviations were higher for larger particles) but it was independent from the contact radius (Pearson correlation of -0.0296).

In summary, we attempted to validate the most important parameters of our approach to reference data (see further details in S1 Dataset). At this point a direct comparison to alternative approaches is hard, since previous work made no claims concerning the accuracy towards separate reference data. Furthermore, previous work neither aimed at determination of particle size nor at optimization of the calculation speed.

## 5 Conclusion

We demonstrated an efficient and accurate detection of multiple radial profiles in RICM images. The focus of the work lies on image analysis algorithm development (and its validation) using a gradient analysis of the image and template matching. We used theoretical templates based on basic physical foundations of RICM microscopy with minor adaptation for known deviations [4]. Our new method always chose the real template position as top match, but sometimes additional false positives were detected. The position is detected precisely (deviation of 1.08 pixels compared to reference data) and the determination of the particle diameter delivers results within a mean error of 2.6 μm for particles with an average diameter of 30 μm. Although template matching is computationally expensive, we achieve relatively quick processing times (<2.5 s per image) thanks to the pre-processing step. Our approach also delivers implicit data about the detected objects derived from the best-matching template. These values include particle radius and contact radius of the SCP and they are of importance for determining adhesion energies between the SCP and its underlying surface in physicochemical, biomedical and biosensing applications.

## Supporting information

S1 Dataset Numerical data from the evaluation of the particle position, particle size, and contact area. (ZIP)
