# Supplementary material for: Radial profile detection of multiple spherical particles in contact with interacting surfaces
Source: PLoS One. 2019 Apr 2;14(4):e0214815. doi: 10.1371/journal.pone.0214815 (PMC6445513; doi:10.1371/journal.pone.0214815)
Supplement: S1 Dataset — (ZIP) [file pone.0214815.s001.zip › S1_Dataset/Description.pdf]

S1 Dataset: Evaluation data for “Radial profile detection of multiple spherical particles in contact with interacting surfaces”

Johannes Waschke, Tilo Pompe, David Rettke, Stephan Schmidt, Mario Hlawitschka

We evaluated the three most important parameters of our results: particle position, particle radius, contact area.

Particle position
The file “1_Particle position” contains the manually determined positions of the profile, the automatically detected positions, and the differences between both value sets.

Particle radius
The file “2_Particle radius” contains the manually determined diameter of a particle, the automatically detected radius, and the comparison between both value sets. The manually defined diameter was retrieved from bright-field data, in which the edge to edge distance was measured in GIMP.

Contact area
The first file (“3_a_Output...”) contains selected output of our software for 205 test cases. It can be seen that 184 particles have a valid contact radius. These have been compared to 40 different settings for region growing (file “3_b_Evaluation...”), where in each case linear regression was performed, the absolute difference was calculated, and correlations were calculated. In the main text, we focus on the setting of 30% for region growing.
